# Functional glyco-metagenomics elucidates the role of glycan-related genes in environments

**DOI:** 10.1186/s12859-021-04425-9

**Published:** 2021-10-18

**Authors:** Hayato Takihara, Nobuaki Miura, Kiyoko F. Aoki-Kinoshita, Shujiro Okuda

**Affiliations:** 1 Division of Bioinformatics, Niigata University Graduate School of Medical and Dental Sciences, 1-757 Asahimachi-dori, Chuo-ku, Niigata, 951-8510 Japan; 2 Glycan and Life Systems Integration Center, Faculty of Science and Engineering, Soka University, 1-236 Tangi-machi, Hachioji, Tokyo 192-8577 Japan

**Keywords:** Functional glyco-metagenomics, Metagenome, Microbiome, Environment, Glycan-related genes, Glycan

## Abstract

**Background:**

Glycan-related genes play a fundamental role in various processes for energy acquisition and homeostasis maintenance while adapting to the environment in which the organism exists; however, their role in the microbiome in the environment is unclear.

**Methods:**

Sequence alignment was performed between known glycan-related genes and complete genomes of microorganisms, and optimal parameters for identifying glycan-related genes were determined based on the alignments. Using the constructed scheme (> 90% of identity and > 25 aa of alignment length), glycan-related genes in various environments were identified from 198 different metagenome data.

**Results:**

As a result, we identified 86.73 million glycan-related genes from the metagenome data. Among the 12 environments classified in this study, the percentage of glycan-related genes was high in the human-associated environment, suggesting that these environments utilize glycan metabolism better than other environments. On the other hand, the relative abundances of both glycoside hydrolases and glycosyltransferases surprisingly had a coverage of over 80% in all the environments. These glycoside hydrolases and glycosyltransferases were classified into two groups of (1) general enzyme families identified in various environments and (2) specific enzymes found only in certain environments. The general enzyme families were mostly from genes involved in monosaccharide metabolism, and most of the specific enzymes were polysaccharide degrading enzymes.

**Conclusion:**

These findings suggest that environmental microorganisms could change the composition of their glycan-related genes to adapt the processes involved in acquiring energy from glycans in their environments. Our functional glyco-metagenomics approach has made it possible to clarify the relationship between the environment and genes from the perspective of carbohydrates, and the existence of glycan-related genes that exist specifically in the environment.

**Supplementary Information:**

The online version contains supplementary material available at 10.1186/s12859-021-04425-9.

## Background

Genome sequencing has been carried out for the past two decades with respect to unicellular and multicellular microorganisms, as well as microbial communities isolated from a variety of environments, including the ocean [1, 2], soil [3], animal digestive tracts [4], and humans [5, 6]. Metagenomics elucidates the nature of life through the exploration of the genetic content of various bacteria from the reads of samples taken from different environments [7–11]. The issue at present is not to obtain more sequence data, but to infer the functions of the myriad of proteins already identified [12]. Although next-generation sequencing has led to an explosion of sequence data, our functional understanding of the data is still lacking [11, 13]. This bias is caused by the lack of diverse lineages and genetic compositions of the microbiome, basically due to the fact that genome sequences registered in databases tend to be biased toward culturable species.

Functional metagenomics is known as a method to search for functional genes in microbial communities based on the results of metagenomic sequencing and experimental screenings [14, 15]. Functional metagenomics has been mostly conducted on intestinal bacteria, it is used to infer what kind of phenomenon in the environment by associating metagenome information related to disease with known metabolic information [7, 8]. Databases that organize information on various functional genes in a broad range of species [16–20] have been constructed, which are more enhancing functional metagenomics. Such functional metagenomics have revealed that an abundance of glycan-related genes reside in the intestinal environment, identifying 95 of 124 (77%) carbohydrate hydrolase families [16, 21, 22]. Since glycan-related genes play a key role in energy acquisition, cell–cell interactions, molecular recognition, signaling transduction, etc. [23], of various organisms, it is considered to be an essential target in terms of its ecological significance. Information on genes involved in polysaccharides and glycans in individual species have drastically increased [22, 24], but functional information about such genes found in environments is still unknown.

Enzymes that assemble and degrade glycans have been classified into sequence-based families by Henrissat et al*. *[25–30] The functional diversity or specificity of these enzymes is enormous and reflects the wide variety of glycan structures found in nature. CAZy is a database that was launched in 1991[25] which collects and organizes information based on these data. Carbohydrate active enzymes registered in CAZy are called carbohydrate-active enzymes (CAZymes). The classification system in the CAZy database is based on the results of biochemical experiments, and genes are classified into specific categories, including glycoside hydrolase (GH), polysaccharide lyase (PL), glycosyltransferase (GT), carbohydrate-binding module (CBM), carbohydrate esterase (CE), as well as several other categories of genes that act on carbohydrates (AA)[22].

The purpose of this study is to elucidate carbohydrate metabolism such as energy acquisition by taking advantage of functional metagenomics and observing an overview of CAZymes (glycan-related genes) in various environments. Using information of metagenome reads in various environment, we found that the environment influences the abundance and types of glycan-related genes, suggesting that their functional roles could be adapted to their environmental glycan characteristics. From these findings, we propose a model for the use of glycan-related genes for energy acquisition of microorganisms in the environments. The functional glyco-metagenomics established in this study can make it possible to infer the role of glycan-related genes and microorganisms in the environment.

## Results

### Evaluation of the accuracy of the identified glycan-related genes based on complete genomes

**Fig. 1:**
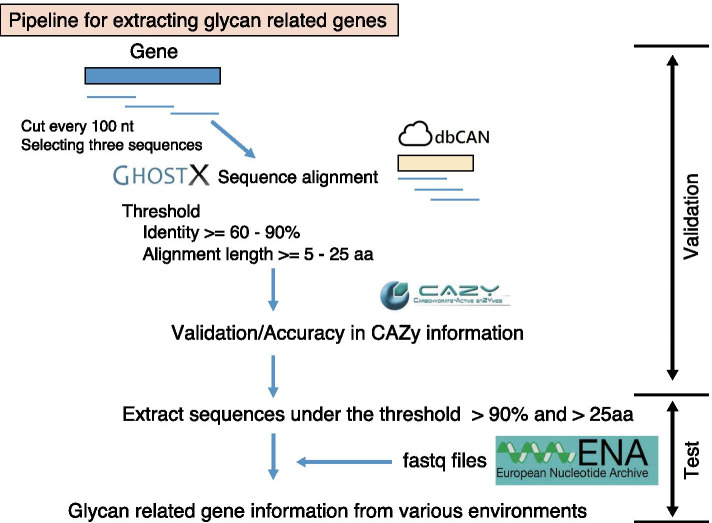
A strategy of identification of glycan-related genes in environments

**Fig. 2:**
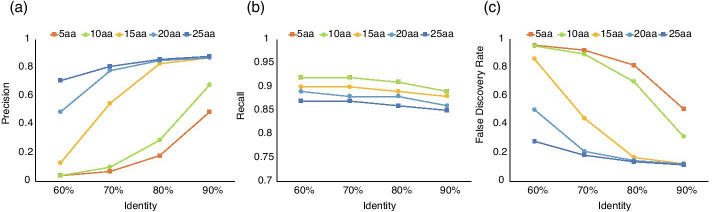
Accuracy assessment of glycan-related gene identification using short read alignments. Accuracy indices used in this study are precision (**a**), recall (**b**) and false discovery rate (**c**)

To obtain information on the distribution of glycan-related genes in the environment, we developed a method to identify glycan-related genes from metagenomic data that are comprehensively sequenced by next-generation sequencers to determine the DNA sequences in the environment (Fig. 1). Metagenomic sequences often consist of short reads of 100–200 bp, and we evaluated the conditions for identifying glycan-related genes based on the sequence homologies of these short reads when aligned against a database of known glycan-related gene sequences. First, we generated virtual shotgun metagenomic data by randomly fragmenting the genomic sequence of 39 completely sequenced genomes that differ at Genus level including 17 Gram-positive bacteria and 22 Gram-negative bacteria (see Additional file 2: Table S1). These virtual metagenomic reads were aligned with 820,000 protein sequences registered in dbCAN [24] as a reference database using GhostX [31]. The alignment results were divided into positive and negative groups based on certain cutoff values using two variables, alignment identity (60–90%) and length (5–25 aa). For the evaluation of prediction accuracy, genes registered in CAZy were used as the correct set, and if a candidate gene was above a certain cutoff value in terms of alignment identity (60–90%) and length (5–25 aa), it was considered true, and otherwise false. The accuracy of glycan-related genes extracted in each bacterial genome was evaluated using Precision, Recall, and FDR (Fig. 2). These results showed that the highest accuracy was obtained when the identity was > 90% and the alignment length was > 25 aa, where Precision was > 90% (Fig. 2a), Recall was < 10% (Fig. 2c) and FDR was < 10% (Fig. 2b). Therefore, this condition was used for the subsequent identification of glycan-related genes.

### Glycan-related genes identified from metagenome data

To investigate the distribution of glycan-related genes in microbial communities in various environments, we used the above method to detect glycan-related genes in the actual environmental metagenome (Fig. 1). We identified 86.73 million glycan-related genes from 198 metagenomic data obtained from ENA [32] (see Additional file 2: Table S3). The ratio of the number of identified glycan-related genes to the total number of reads included in each metagenomic data was high in the human-related environment such as human intestine and oral cavity, but 0.01% or less in the remaining 19 metagenome data (eleven from the deep sea, two from a hot spring, five from oil contamination, and one from rhizosphere).

**Fig. 3:**
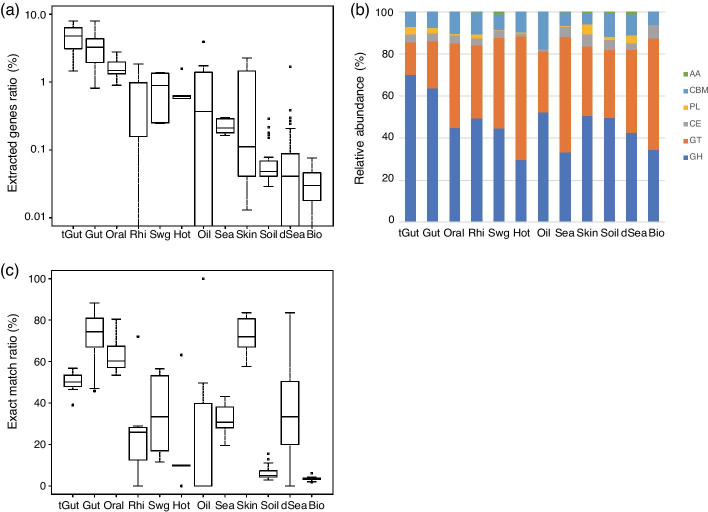
Glycan-related genes identified from environmental metagenome sequences. **a** Ratio of glycan-related genes in each environment. To calculate the ratio, the number of reads mapped to glycan-related genes was divided by the total number of reads. **b** Relative abundance of enzyme classes of CAZy in the identified glycan-related genes. *GH* glycoside hydrolase, *GT* glycosyltransferase, *CE* carbohydrate esterase, *PL* polysaccharide lyase, *CBM* carbohydrate binding module, *AA* auxiliary activities. **c** Ratio of glycan-related genes perfectly matched with the sequence stored in the dbCAN database. The body of box plots in (**a**) and (**c**) goes from the first to third quartiles of the distribution and the center line is at the median. The abbreviation of the environmental names are *Bio* biofilm, *dSea* deep sea, *Gut* gut, *Hot* hot spring, *Oil* oil contaminated, *Oral* oral, *Rhi* rhizosphere, *Sea* sea, *Swg* sewage, *Skin* skin, *Soil* soil, *tGut* tumor gut

In addition, the identified glycan-related genes differed greatly in their proportions in the environment compared to individual metagenomes. Thus, we calculated the distribution of the coverage of glycan-related genes in each environment (Fig. 3a). As a result, glycan-related genes were identified more in the human-associated metagenomes (average 2.64%) consisting of Gut (0.8–8.0%), Oral (0.9–2.8%), tGut (1.5–8.5%) and Skin (0.01–2.1%) than those in other environments (average 0.27%).

Although the number and content of glycan-related genes in the environment suggest that they are affected by the environment, their roles in each sample is unknown because glycan-related genes have a high variety of functions, from hydrolysis to transfer reactions of glycosidic bonds between sugars. Therefore, the identified glycan-related genes were annotated with the six functional categories defined by CAZy. The functions of the identified glycan-related genes were classified using the sequence annotations of the top hits. In each metagenome data set, we calculated the ratio of the six categories in which all of the identified glycan-related genes were annotated (Fig. 3b, Additional file 2: Table S4). We found that the proportion of the six classifications showed little difference among the samples. However, the ratio of GH (glycoside hydrolase) and GT (glycosyltransferase) in each environment was very high at 40–60%, and, surprisingly, the combined ratio of both was constantly over 80% in all environments. There are six environments with the highest number of GH (Gut, tGut, Rhizosphere, Oil contamination, Skin, Soil) and six environments with the highest number of GTs (Oral, Sewage, Hot spring, Sea, Deep sea, Biofilm). In particular, the relative amount of GT was highest in Hot spring, Sea, and Biofilm. Thus, GTs are expected to be important in such aquatic feature and biome. From these results, it is possible that the ratio of the functions of glycan-related genes differs depending on the environment, and that the functions of the assembly of these genes also differ depending on the environment.

In this study, > 90% identity was used as a parameter to identify novel glycan-related genes. To examine the extent to which known glycan-related genes in the environment were identified, we calculated the percentage of amino acid sequences that completely matched the amino acid sequences in the reference database we used (Fig. 3c, Additional file 2: Table S5). Sequences that exactly matched the sequences registered in dbCAN varied among the metagenomic samples. However, when compared by environment, Gut, Skin, and Oral had a large number of exact matches, exceeding 50%, whereas Soil had a low number of less than 10%. This suggests that human-related environments, such as Gut and Skin, are often well-studied as research subjects, indicating that a large number of CAZymes are registered from these environments.

### Enrichment of GH and GT family in various environments

**Fig. 4:**
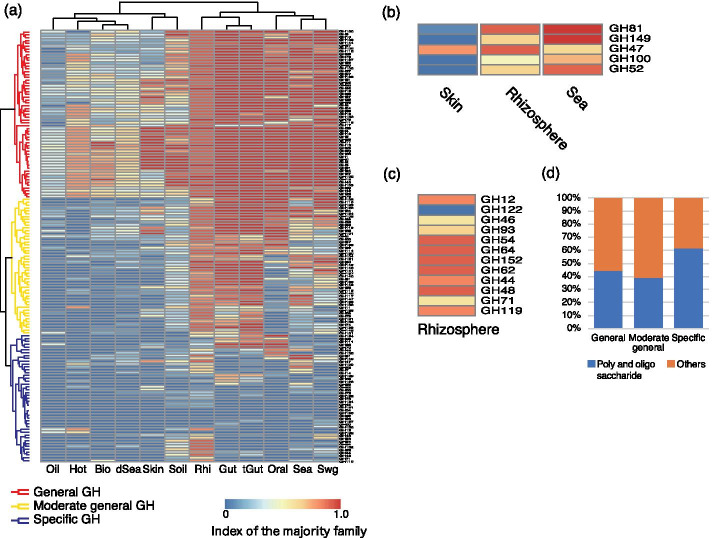
Clustering of the families and distribution of the substrates in GH. **a** Cells in the heatmap show the *I*_ms_ of GH families (see Methods). **b** Remarkable family of specific-GH in skin, rhizosphere and sea. This figure is an enlargement of (**a**). **c** Remarkable family of specific-GH in rhizosphere. This figure is an enlargement of (**a**). **d** Ratio of the number of families that avail polysaccharide and oligo-saccharide as substrates among all GH families. The abbreviation of the environmental names are *Bio* biofilm, *dSea* deep sea, *Gut* gut, *Hot* hot spring, *Oil* oil contaminated, *Oral* oral, *Rhi* rhizosphere, *Sea* sea, *Swg* sewage, *Skin* skin, *Soil* soil, *tGut* tumor gut

Since carbohydrates are a source of energy for many microorganisms, their glycan-related genes may be adapted to different types and compositions of carbohydrates in their environment. Here, we defined *I*_*ms*_ as the proportion of metagenomic samples in the environment in which glycan-related genes for a family have been identified (see Methods). First, for the GH families, we examined the distribution of each family in the environment. We performed clustering analysis in order to classify the distribution of GH families in each environment (Fig. 4, Additional file 2: Table S6). As a result, we found that there are GH families specific to an environment, and therefore, GH families can be classified into several groups depending on the pattern of GH distribution. The family of GHs commonly detected in most of the environments, those in a few environments, and those in between were designated general-GH, specific-GH, and moderate general-GH, respectively (Fig. 4a). The general-GH group contained enzymes such as α-amylase found in many species. However, rare sugar hydrolytic enzymes such as xylanase and fucosidase were found in moderate general-GH, whereas polysaccharide-degrading enzymes such as glucanase and chitinase were found in specific-GH. Specific and moderate general families showed high *I*_ms_ in the rhizosphere (> 0.6 of *I*_ms_), whereas most of the others showed lower values in oil contamination samples (< 0.3 of *I*_ms_).

As a distinctive family of specific-GH, α-mannosidase (GH47) showed high *I*_ms_ (> 0.7 of *I*_ms_) in skin, sea, and rhizosphere (Fig. 4b). This is an enzyme that trims high- (oligo-) mannose type *N*-glycans, and it is unique to eukaryotes. Therefore, it was considered to be a gene derived from yeast and fungi in the environment [33]. In Rhizosphere, a group of GHs showing high *I*_ms_, endoglucanase (GH44, GH64, GH152), chitinase (GH48), xylosidase (GH54), α-L-arabinofuranosidase (GH62), nigeran digestion enzyme (GH71), and α-Amylase (GH119) were found (Fig. 4c). These are polysaccharide-degraders, which are thought to originate from species living in the plant-derived polysaccharide-rich rhizosphere [34, 35].

To investigate role of specific-GHs in each environment, based on the GH family information in CAZy, the substrates of each family were classified into polysaccharides, oligosaccharides, disaccharides and monosaccharides. The fraction of polysaccharide and oligosaccharide families in specific-GH was 61%, whereas that of general-GH was 44%, and moderate-GH was 39% (Fig. 4d, Additional file 2: Table S8). This fraction in the specific-GH group was significantly higher than in general-GH (p = 0.01, Chi-squared test) and moderate-GH (p = 0.03, Chi-squared test). GH is a hydrolytic enzyme, an enzyme that builds the metabolic system for energy acquisition mainly through the degradation of sugars. This suggests that these enzymes break down polysaccharides specific to each environment into small sugars to obtain energy.

**Fig. 5:**
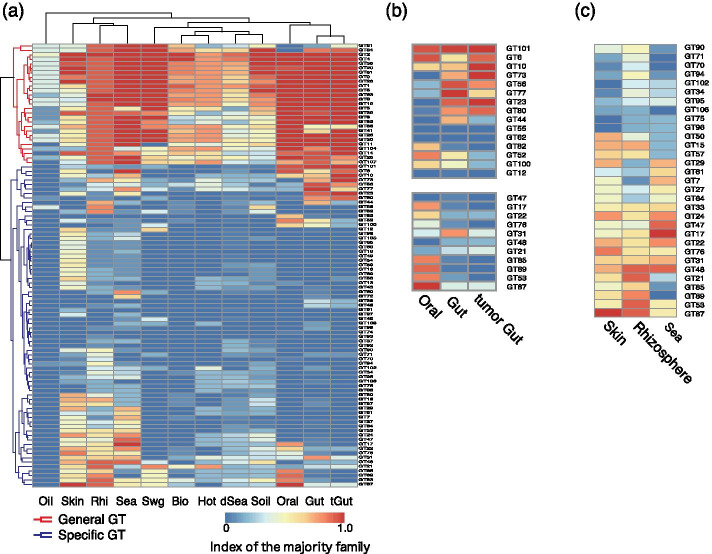
Clustering of the families and distribution of the substrates in GT. **a** Cells in the heatmap show the *I*_ms_ of GT families (see Methods). **b** Remarkable family of specific-GT in oral, gut and tumor gut. This figure is an enlargement of (**a**). **c** Remarkable family of specific-GT in skin, rhizosphere and sea. This figure is an enlargement of (**a**). The abbreviation of the environmental names are *Bio* biofilm, *dSea* deep sea, *Gut* gut, *Hot* hot spring, *Oil* oil contaminated, *Oral* oral, *Rhi* rhizosphere, *Sea* sea, *Swg* sewage, *Skin* skin, *Soil* soil, *tGut* tumor gut

Next, we performed clustering analysis in order to classify the distribution of GT families in each environment (Fig. 5, Additional file 2: Table S7). Two groups of GT types that are specific to each environment were detected based on their pattern of GT distribution (see Additional file 2: Table S9). The family of GTs commonly detected in 11 environments including 170 metagenomes was designated general-GT, and those in specific environments were designated specific-GT. A number of GTs such as trehalose phosphatase were found in many species in the general-GT group and specific enzymes such as mannosyl-transferase in the specific-GT group.

As a characteristic example of specific-GTs, there was a group of inverted *I*_ms_ pattern between gut and oral (Fig. 5b). The species harboring families showing high *I*_ms_ in Oral (> 0.7 of *I*_ms_), such as α-L-arabinosyltransferase (GT53), α-D-arabinofuranosyltransferase (GT85), α-1,2-mannosyltransferase (GT87), and β-1,2-arabinofuranosyltransferase (GT89), were investigated. As a result, it was found that α-L-arabinosyltransferase (GT53) is an enzyme widely identified in prokaryotes, while galactane α-D-arabinofuranosyltransferase (GT85), α-1,2-mannosyltransferase (GT87), and β-1,2-arabinofuranosyltransferase (GT89) are enzymes identified in *Corynebacterium* and *Mycobacterium*. These microbial species can indeed be detected in oral and the nasopharynx, and the present results support this fact [36–38].

Regarding the five GT families with high *I*_ms_ in gut (> 0.7 of *I*_ms_), α-1,6-l-fucosyltransferase (GT23), α-glucosyltransferase (GT44), Fuc4NAc transferase (GT56), α-1,3-galactosyltransferase (GT77), α-2,6-sialyltransferase (GT80), their species were investigated. It was found that α-glucosyltransferase (GT44) was identified in *Chlamydia* and pathogen-*Escherichia*, Fuc4NAc transferase (GT56) in *Salmonella* and *Klebsiella*, and α-2,6-sialyltransferase (GT80) in Pasteurellaceae and *Citrobacter*. In addition, α-1,6-l-fucosyltransferase (GT23) has been identified in *Bacteroides fragilis* [39]. More than 90% of the species in these families registered on CAZy were species known to reside in the gut microbiome. It was reported that GT77 inactivated in the human intestine is carried by a group of microorganisms such as *Streptococcus* and *Escherichia* [40]. These results indicate that the CAZy families of gut and oral with reversed *I*_ms_ patterns is due to the species in the environment.

β-glucosyltransferase (GT21) showed high *I*_ms_ in rhizosphere (> 0.7 of *I*_ms_), sea (> 0.4 of *I*_ms_), sewage (> 0.7 of *I*_ms_), and hot spring (> 0.7 of *I*_ms_), which is an enzyme involved in pullulan synthesis and is known to be possessed by the genus *Aspergillus* [41]. β-1,4-*N*-acetylglucosaminyltransferase (GT17, MGAT3) has been identified in all metagenome samples of sea and in species such as *Acetobacter* and *Enterobacter* found in the ocean [42, 43].

## Discussion

For functional metagenomics, it is desirable that databases used for gene annotation have sufficiency and little bias. In this study, strict search results showing complete homology suggest that many genes registered in CAZy and dbCAN were detected in human-related environments. Bias in the human-related environment as reference gene sequences used for gene function prediction could lead to missed prediction or mass production of hypothetical proteins. If the search is based on exact match only, this bias towards gene sequence data from the human-related environment is likely to have a significant impact on the search results. However, we did not take this bias into account in the present analysis because the search results with the method we developed in this study (> 90% identity, > 25 amino acids alignment) showed sufficiently high accuracy in searching for homology with glycan-related genes.

For example, the number of glycan-related genes identified in the soil environments increased in more samples using our method than in the exact match criterion. An environment in which such new genes are often found is very likely to be a candidate environment for new gene discovery. In addition, the number of entries in CAZy and dbCAN has been increasing every year, and new families of each classification for new reaction modes and substrates continue to be established. GH157-161 was newly established in 2019, and GHs related to that family accounted for an average of 0.26% (0–2.6%) of the GHs identified in the present results. Therefore, it is highly possible that novel glycan-related genes with different substrates and functions could be identified in the soil environment, compared with the case of using the reference sequence database before that. Thus, it is highly likely that the opportunities for the discovery of novel glycan-related genes will increase as CAZy continues to be updated in the future.

The proportion of identified glycan-related genes between the environments showed a slight difference in intra-environments, but a large difference in inter-environments (see Additional file 1: Fig. S1). In particular, the proportion of the identified genes was higher in the human-associated environments, which is consistent with previous studies showing that the microbiome in human-associated environments harbor more genes for sugar-based metabolic systems (e.g., energy production) [16, 21, 22]. On the other hand, deep sea and oil contamination etc. detected a lower proportion of glycan-related genes, but in such an oligotrophic environment, there is less opportunity for sugar to be supplied to the environment due to less material circulation. Therefore, it is possible that the lower need for glycan-related genes in these environments compared to other genes may have led to the adaptation to a lower proportion of glycan-related genes.

Furthermore, the pattern of distribution was divided into two groups, one with a high GH content and the other with a high GT content. Surprisingly, the sum of both GHs and GTs remained constant at 80%, despite the fact that the ratio of the identified glycan-related genes differed between environments. Since the state of sugars may differ depending on an environment, gene organization in microbial species could be adaptable to substances in the environment. Specific enzymes are considered to play a role in the adaptation to the environment. In this study, we explored glycan-related genes, but genes related to other substances (organic compounds such as lipids and phosphate groups) may also show similar trends. Oil degrading bacteria such as *Pseudomonas* and *Rhodococcus* exist in the oil environment and can derive their energy from petroleum hydrocarbons by enzymes such as alkane hydrogenase [44, 45]. This suggests that microbes in the Rhizosphere may derive their energy from sugars, whereas in the oil environment they may derive it from oil.

General glycan-related genes for both GH and GT families were widely distributed in various environments. When these genes were mapped to "Starch and sucrose metabolism" in the KEGG PATHWAY [46–48], they accounted for 68% of the 76 EC numbers on that pathway (see Additional file 2: Table S10). From the mapped genes on the pathway, a series of metabolisms were linked from Amylose and GlcNAc to Glycolysis via Glucose, Xylose, Arabinose and Mannose. Therefore, general-GH and general-GT could play an important role in energy acquisition. In the case of the genes in the specific-GH and specific-GT families, no enrichment to certain pathway maps was detected. Thus it can be expected that these genes are most likely involved in a specific metabolic pathway in each environment, rather than a common pathway across environments.

**Fig. 6:**
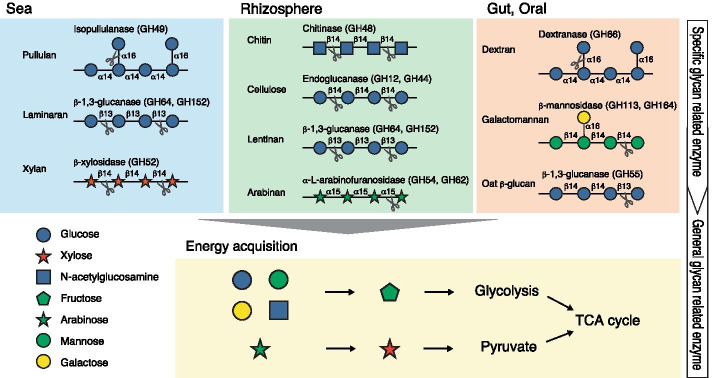
A universal model of the roles of general and specific glyco-enzymes in environments for general and specific enzymes

Among these specific-enzymes, many were enzymes that decompose polysaccharides. Certain distinctive specific enzymes were found such as plant-derived polysaccharides [49] (chitin, cellulose, lentinan, xylan) in rhizosphere, pullulan and laminaran in sea, and dextran and galactomannan in gut and oral [50]. These environment-specific glycans could be decomposed into more general glycan components by the specific enzymes in each environment. This would allow them to be further decomposed by a more common pathway of glycan metabolism. Finally, we surmise that general-enzymes play a role in the acquisition of energy from these glycan components (Fig. 6). According to this universal energy acquisition model, it is possible that environmentally-specific species and genes may play a role in regulating sugar metabolism in the environment.

## Conclusions

It is important to evaluate the environment of the sample by predicting the function from metagenomic reads and inferring phenomena occurring in the sample. Our method should play an important role in establishing functional glyco-metagenomics to identify glycan-related genes in the environment from metagenomic data and to hypothesize the role of sugars by comparing them within and between environments. In addition, although we performed our analysis using metagenomic data already registered in repository databases, our re-analysis was able to illustrate new perspectives regarding sugars in the target environment. By applying our methods, we hope to find new perspectives and discoveries as we can now reanalyze large amounts of historical metagenomic data for comparison in various environments.

## Methods

### Validation of identification glycan-related genes using sequence alignment

We developed a method for identifying glycan-related genes from nucleotide sequences such as short read sequences obtained from NGS using sequence alignment. The microbial genome of 39 genera (see Additional file 2: Table S1) belonging to multiple phyla was downloaded from KEGG [46–48]. Since these contained gene annotations, glycan-related genes could be found by referencing CAZy (http://www.cazy.org). If a detected gene was a glycan-related gene, the label “True” was added to the gene data, otherwise, the label “False” was added. The DNA sequences were cut every 100 bases from the 5’ end, and three fragments were randomly selected for each gene. This process was performed with our ad hoc ruby scripts. The sequences of these fragments were converted to amino acid sequences using the standard genetic code. Six possible amino acid sequences were generated from a single fragment, due to the six frames possible on the forward and reverse strands and starting DNA position for each codon. This was used as the model dataset for evaluation of our method to identify glycan-related genes from metagenomic sequence data. The fragment peptide sequences obtained in these processes were generated using GhostX version 2.1 [31].

The 1.38 million amino acid sequences of proteins in FASTA format with respect to glycan-related genes were downloaded from the dbCAN meta server (http://bcb.unl.edu/dbCAN2/). After deleting approximately 550,000 redundant sequences, the resulting database consisted of approximately 830,000 sequences, for the reference data to identify glycan-related genes.

The alignments were carried out between all fragment peptide sequences and amino acid sequences of the reference database using GhostX. It is expected that in the case of glycan-related genes, alignments show high identity and long alignment length. Therefore, prediction of whether each fragment was a glycan-related gene was performed under the conditions of identity thresholds of 60–90% and an alignment length threshold of 5–25 aa using a validation dataset where each fragment is known to be glycan-related gene. The effectiveness of our prediction method was evaluated by computing the precision, recall and false discovery rates which were calculated according to following equations:

$$\begin{aligned} & {\text{Precision}}\, = \,{\text{True}}\,{\text{Positive/}}\left( {{\text{True Positive}}\, + \,{\text{False}}\,{\text{Positive}}} \right) \\ & {\text{Recall}}\, = \,{\text{True}}\,{\text{Positive/}}\left( {{\text{True}}\,{\text{Positive}}\, + \,{\text{False}}\,{\text{Negative}}} \right) \\ & {\text{FDR}}\, = \,{\text{False}}\,{\text{Positive/}}\left( {{\text{True}}\,{\text{Positive}}\, + \,{\text{False}}\,{\text{Positive}}} \right) \\ \end{aligned}$$

for each genome.

### Acquisition and sequence alignment of metagenomes in various environments

In total, 198 metagenomes were downloaded from the European Nucleotide Archive (https://www.ebi.ac.uk/ena). Each of these include over two million reads whose lengths are over 100 bases. Additional file 2: Table S2 shows the accession numbers, the environments from which the genomes were taken, and the total number of sequence reads. These 198 metagenomes were classified into 12 environments based on the Metagenome/Microbe Environmental Ontology [51]. According to the method described in the previous section, sequence alignment of metagenome reads was performed using GhostX.

### Identification of glycan-related genes and organization of gene information

For our 198 metagenomes, the populations of the reads of glycan-related genes against the total number of reads were calculated, and the averages and the standard deviations of the populations of glycan-related genes in the metagenomes were calculated for each of the 12 environments. The relative population of each class of CAZy against the glycan-related genes were also calculated.

The identification of glycan-related genes is based on > 90% of identity and > 25 aa of alignment length thresholds. This method was used as the optimal condition to search for a wide variety of glycan-related genes. However, it is not possible to distinguish whether the identified genes by our method are known genes or undiscovered genes. Of these identified glycan-related genes, genes showing 100% identity to the reference sequences were explored. Since these exact-matched sequences are no different from known gene sequences, at least with respect to their target sequence regions, they were considered as genes already identified and registered in the public databases. The processes in this section to identify glycan-related genes from the GhostX results were performed with our ad hoc ruby scripts.

### Comparison of glycan-related genes for each environment

According to the CAZy functional classification, the glycoside hydrolase (GH) and the glycosyltransferase (GT) families, were classified into 166 and 109 families, respectively. The obtained reads of glycan-related genes were thus also classified by family. We defined the index of the majority family (*I*_ms_) in an environment as the ratio of the number of metagenomes with the identified glycan-related genes to the number of metagenomes belonging to the environment. *I*_ms_ indicates how major the family of the glycan-related genes is in a given environment. If *I*_ms_ = 1, it means that all metagenomes in the environment have glycan-related genes in the family, whereas *I*_ms_ = 0 means that there are no glycan-related genes in the family. This process was performed using ad hoc ruby scripts. In order to visualize this data across each environment and family, Euclidean distances were calculated and hierarchical clustering analysis was carried out using the Ward’s method with the pheatmap library (https://cran.r-project.org/web/pachkages/pheatmap/index.html) for R (https://www.R-project.org/) was used to produce the heatmap figure with the default color setting for this clustering.

The families of GHs were also classified according to substrate specificities, which are also in the CAZy database. The families of GHs were classified into monosaccharides, disaccharides, oligosaccharides, polysaccharides, peptidoglycan-related, and others, based on the substrate information provided by the CAZy database and the more detailed descriptions in KEGG COMPOUND [46–48]. In order to analyze the functions of the genes in the general-enzyme families, the EC numbers of general-enzyme genes were mapped to “Starch and sucrose metabolism” in the KEGG PATHWAY maps, and the mapped EC numbers were counted.

## Supplementary Information

**Additional file 1: Fig. S1**. Percentage of predicted glycan-related genes in each metagenomic sample. The abbreviation of the environmental names are Bio: biofilm, dSea: deep sea, Gut: gut, Hot: hot spring, Oil: oil contaminated, Oral: oral, Rhi: rhizosphere, Sea: sea, Swg: sewage, Skin: skin, Soil: soil, and tGut: tumor gut.

**Additional file 2: Table S1**. List of bacterial taxonomy used in this study. **Table S2**. List of metagenomic samples used in this study. **Table S3**. Glycan-related genes predicted in each metagenome sample. **Table S4**. CAZy classification of predicted glycan-related genes in each metagenome sample. **Table S5**. Glycan-related genes perfectly matched to the dbCAN sequences. **Table S6**. GH families in each metagenome sample. A cell value is relative abundance in each sample. **Table S7**. GT families in each metagenome sample. A cell value is relative abundance in each sample. **Table S8**. Classification of substrates and properties of GH families. **Table S9**. Classification of specific- or general-properties of GT families. **Table S10**. Coverage of EC numbers on the KEGG pathway map “Starch and sucrose metabolism” (KEGG PATHWAY ko00500).

## Data Availability

All data generated or analyzed during this study are included in this published article and its supplementary information files. All sequence files used publicity available data in European Nucleotide Archive. Accession numbers were showed Additional file 2: Table S2.
