# Supplementary figures and images for: Functional glyco-metagenomics elucidates the role of glycan-related genes in environments
Source: BMC Bioinformatics. 2021 Oct 18;22:505. doi: 10.1186/s12859-021-04425-9 (PMC8522060; doi:10.1186/s12859-021-04425-9)

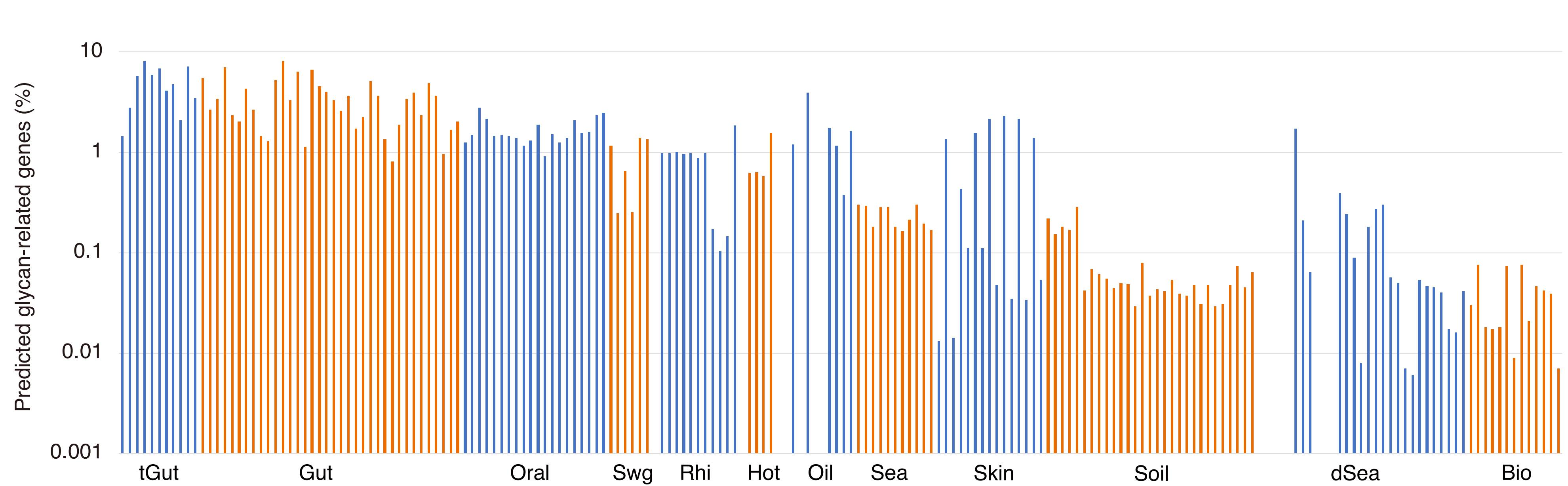

Supplement: Supplementary file 1 — Additional file 1: Fig. S1. Percentage of predicted glycan-related genes in each metagenomic sample. The abbreviation of the environmental names are Bio: biofilm, dSea: deep sea, Gut: gut, Hot: hot spring, Oil: oil contaminated, Oral: oral, Rhi: rhizosphere, Sea: sea, Swg: sewage, Skin: skin, Soil: soil, and tGut: tumor gut. [file 12859_2021_4425_MOESM1_ESM.pdf]
